# The relationship between serum levels of LOX-1, hs-cTnT, NGAL, and renal function, and their diagnostic value in patients with chronic kidney disease: a retrospective study

**DOI:** 10.1186/s12882-024-03875-6

**Published:** 2024-11-27

**Authors:** Liyin Chai, Jun Zeng, Li Gong, Zhuhong Li, Fang Wang, Zhengyang Liu, Wang Fan, Bingbing Shen

**Affiliations:** 1 Department of Nephrology, Chongqing Emergency Medical Center, Chongqing University Central Hospital, No.1 Health Road, LiangLukou, Yuzhong District, Chongqing City, 400014 China; 2 Chongqing Emergency Medical Center, School of Medicine of Chongqing University, Chongqing University Central Hospital, Chongqing City, China; 3 Chongqing Hechuan Huatan Hospital, Chongqing City, China

**Keywords:** Chronic kidney disease, Renal function, LOX-1, hs-cTnT, NGAL

## Abstract

**Background:**

The primary aim of this study is to explore the relationship between serum levels of LOX-1, hs-cTnT, and NGAL, and renal function in patients with CKD, as well as to evaluate their diagnostic value for early detection and monitoring of disease progression in CKD patients.

**Methods:**

A retrospective study was conducted on 108 patients with chronic kidney disease admitted to our hospital from January 2023 to December 2023. The patients were divided into the mild renal insufficiency group (51 cases) and the severe renal insufficiency group (57 cases). The differences in serum levels of LOX-1, hs-cTnT, and NGAL between the two groups were compared, and Pearson correlation analysis was used to explore the relationship between the three levels and renal function. ROC analysis was used to evaluate the predictive value of the three markers for the diagnosis of CKD.

**Results:**

The levels of LOX-1, hs-cTnT, and NGAL in the mild renal insufficiency group were lower than those in the severe renal insufficiency group (*P* < 0.05). Correlation analysis showed that serum levels of LOX-1, hs-cTnT, and NGAL were positively correlated with the deterioration of renal function (*P* < 0.001), indicating a significant correlation between LOX-1, hs-cTnT, NGAL levels, and the deterioration of renal function. ROC analysis showed that the AUC of serum levels of LOX-1, hs-cTnT, and NGAL were 0.859, 0.882, and 0.841, indicating a significant predictive value for the diagnosis of chronic kidney disease.

**Conclusion:**

Serum levels of LOX-1, hs-cTnT, NGAL, and related markers demonstrate a direct association with the extent of renal impairment, offering predictive capabilities for diagnosing CKD.

## Introduction

Chronic kidney disease (CKD) is a significant and prevalent clinical condition in China, affecting up to 10.8% of the population and with a prevalence of 23.4-35.8% among individuals over 64 years old [1]. Despite some advancements in CKD prevention and treatment, the complex causes and unclear mechanisms of the disease have hindered the development of effective preventive and therapeutic strategies. Early diagnosis, timely treatment, and accurate prognosis assessment are crucial for improving CKD patient survival rates [2]. CKD can be caused by various factors, including hypertension, diabetes, and acute kidney injury [3]. The pathological changes induced by different causes can lead to oxidative stress, inflammatory damage, renal anemia, and other pathological alterations in intrarenal and extrarenal cells, accelerating CKD progression to end-stage renal disease [4]. Studying the causes and related risk factors of CKD can help identify its development trends and facilitate early diagnosis and treatment.

Currently, common detection methods include measuring glomerular filtration rate (GFR), urinary protein levels, and creatinine levels [5]. However, the stability and reliability of these results can be affected by factors such as anemia and stroke, which can lower the glomerular filtration rate. Therefore, it is necessary to identify reliable markers of kidney damage to assess the occurrence and progression of the disease in patients. Legtin-type oxidized LDL receptor 1 (LOX-1) is a new receptor for oxidized low-density lipoprotein (Ox-LDL), which regulates endothelial cell activation and transformation by binding to Ox-LDL, upregulating the expression of intercellular adhesion molecule 1 (ICAM-1) in endothelial and mesangial cells, and inducing inflammatory reactions [6]. LOX-1 is upregulated in various pathological conditions, including diabetes, coronary artery disease, hypertension, and glomerulosclerosis [7]. Studies have shown high expression of LOX-1 in ox-LDL-induced glomerular podocytes, suggesting that LOX-1 may serve as an important marker of glomerular injury [8]. Neutrophil gelatinase-associated lipocalin (NGAL) is an important inflammatory response factor discovered in recent years and has been widely recognized in clinical practice [9]. Previous studies have found that increased serum NGAL levels in CKD patients can cause or exacerbate anemia caused by CKD, suggesting that NGAL might also serve as an indicator in CKD early diagnosis [10].

CKD patients are also at high risk for cardiovascular disease (CVD), with higher incidence and mortality rates than the general population [5]. Cardiac troponin T (cTnT) is one of the widely used cardiac markers in clinical practice, with high sensitivity and specificity for myocardial injury [8, 11, 12]. The hs-cTnT is an indicator that can reflect early myocardial micro-damage, and its detection in CKD patients can effectively prevent and treat CKD-CVD. The level of hs-cTnT in CKD patients is significantly higher than that in non-CKD individuals, and some scholars believe that this is due to the decrease in renal clearance ability, while others suggest it is related to the presence of CVD in CKD patients [13–15]. Previous studies have reported that the normal range for serum hs-cTnT is typically around < 40 pg/mL in healthy individuals. Similarly, NGAL levels in healthy individuals are usually 80–100 ng/mL, while normal values for LOX-1 in healthy individuals are approximately < 20 ng/mL. Elevated levels of hs-cTnT have been observed in cardiovascular disease patients, particularly during and after myocardial infarction, where values can exceed 300 pg/mL [16–18]. Currently, there are few studies on the relationship between serum levels of LOX-1, hs-cTnT, NGAL, and CKD. Based on this, this study aims to explore the relationship between serum levels of LOX-1, hs-cTnT, and NGAL, and renal function in patients with CKD. Furthermore, the study evaluates the diagnostic value of these biomarkers for early detection and monitoring of disease progression in CKD patients, providing a theoretical basis for the diagnosis and treatment of chronic kidney disease.

## Methods

### Study population

A retrospective study was conducted on the clinical data of 108 patients with chronic kidney disease admitted to our hospital from January 2023 to December 2023. According to the ratio of urinary microalbumin to creatinine (ACR), the patients were divided into microalbuminuria group (51 cases, ACR abnormal: 30-300 mg/g) and proteinuria group (57 cases, ACR highly abnormal: >300 mg/g), namely the mild renal insufficiency group (*n* = 51) and severe renal insufficiency group (*n* = 57). The minimum GFR of patients included in this study was 45 mL/min/1.73 m^2^, and the minimum ACR for proteinuria was 35 mg/g, consistent with the CKD diagnostic criteria. The use of medications such as ACE inhibitors and ARBs, which were utilized by 42% and 37% of patients respectively, is known to provide renal protection in patients with chronic kidney disease. Furthermore, SGLT2 inhibitors were used in 25% of patients. In all patients, the mild renal insufficiency group consisted of 30 males and 21 females, with a mean age of (59.43 ± 6.21) years; the severe renal insufficiency group consisted of 35 males and 22 females, with a mean age of (61.25 ± 8.12) years.

### Inclusion criteria

Inclusion criteria: ①Patients diagnosed with chronic kidney disease according to the Kidney Disease Outcome Quality Initiative (K/DOQI) guidelines [19]; ②Age ≥ 18 years; ③History of chronic kidney disease for more than 6 months; ④Patients with complete clinical data; ⑤Patients who can strictly follow medical advice and receive treatment according to physician’s instructions. Exclusion criteria: ①Patients who do not meet the inclusion criteria; ②Patients who have started dialysis or are expected to undergo dialysis treatment within a short period (≤ 3 months); ③Secondary kidney diseases, such as diabetic nephropathy, hypertensive nephropathy, lupus nephritis, etc.; ④Patients with acute kidney injury or acute renal failure or prior history of acute kidney injury; ⑤Patients with other kidney diseases, such as polycystic kidney disease, solitary kidney, etc.; ⑥Patients with autoimmune diseases, history of corticosteroid use, long-term use of immunosuppressive agents, etc.; ⑦Patients with severe systemic diseases; ⑧Patients with mental disorders; ⑨Patients with incomplete medical records.

### Grouping method

Ten milliliters of midstream urine was collected from both groups of subjects and the urine was centrifuged at 3000 r/min for 10 min before testing and the supernatant was taken. The levels of urinary microalbumin and urine creatinine were measured using a fully automatic specific protein analyzer (A25, BIOSTEC, Spain). Microalbuminuria was measured by immunoturbidimetry and urine creatinine was measured by enzymatic method. The urine analyzer (LX-8000, LONGXIN, China) was used to measure the levels of microalbuminuria (MALB), urine creatinine (UCR), and ACR in each group. Normal ACR: <30 mg/g; ACR abnormal: 30–300 mg/g; ACR highly abnormal: >300 mg/g.

### Laboratory tests

Baseline data: mainly including age, gender, BMI, diabetes, hypertension, cardiovascular disease, smoking history, drinking history, family history of CKD, etc.

LOX-1: 5 mL of fasting venous blood was collected from the research subjects and placed in a centrifuge tube. After centrifugation at 3000 r/min for 10 min, the serum was separated and stored in a -80 ℃ refrigerator. The serum LOX-1 level was measured using an ELISA reader (Anthos 2010, Biochrom, UK) and a kit (ab212161, Abcam, USA).

hs-cTnT: 3 ml of the patient’s venous blood was collected using heparin anticoagulation tubes, and plasma was separated after centrifugation, and hs-cTnT was measured using a fully automated chemiluminescence analyzer (Cobas601, Roche, Germany).

NGAL: 5 mL of fasting venous blood was taken from patients in the morning, and the supernatant was centrifuged at 218×g for 10 min at 4℃. The supernatant was again centrifuged at 4 °C and 706×g for 10 min, collected, and stored at -80 °C. A fully automated biochemical analyzer (Au5800, Beckman Coulter, USA) and a kit (ab113326, Abcam, USA) were used to detect NGAL.

### Statistical analysis

SPSS 26.0 software was used for analysis. Count data were expressed as n (%) and analyzed using the chi-square test. Normally distributed data were expressed as (X ± s) and analyzed using the t-test. If the data were not normally distributed, they were transformed for normal distribution analysis. The correlation of ACR with markers as well as the correlations between the levels of these proteins calculated using Pearson correlation analysis. With ACR highly abnormal as the diagnostic target, the sensitivity and specificity of each marker were calculated and the AUC values were derived. A *P*-value less than 0.05 was considered statistically significant.

## Results

### General data

Comparing the general data of the two groups, there was no statistically significant difference (*P* > 0.05) in age, gender, BMI, diabetes, hypertension, cardiovascular disease smoking history, alcohol consumption history, and family history of CKD between the mild renal dysfunction group and the severe renal dysfunction group (Table 1). This indicates that the groups were relatively homogeneous concerning these factors. However, it does not imply that these factors have no impact on renal dysfunction. Studies have shown that as GFR decreases, urinary protein levels (specifically ACR) tend to increase, indicating an inverse relationship between proteinuria and GFR that reflects progressive glomerular damage and worsening kidney function [20]. To determine their influence on kidney function, further analysis is needed to examine their relationship with GFR in both groups. Therefore, we further analyzed the correlation between various factors and GFR. The results showed that age, diabetes, and cardiovascular disease were significantly negatively correlated with GFR, indicating that these factors may have a significant impact on kidney function decline. Specifically, older age was significantly associated with decreased kidney function (*r* = -0.321, *P* = 0.022), and both diabetes (*r* = -0.273, *P* = 0.041) and cardiovascular disease (*r* = -0.287, *P* = 0.035) were associated with lower GFR values. This suggests that, there were no statistically significant differences in these variables between the mild and severe renal dysfunction groups (*P* > 0.05).

**Table 1:** Comparison of general data between the two groups

	Mild renal insufficiency group (*n* = 51)	Severe renal insufficiency group (*n* = 57)	t/X^2^	*P* value	Correlation with the GFR (*r*)	*P* value
Age (years)	59.43 ± 6.21	61.25 ± 8.12	1.316	0.191	-0.321	0.022
Gender (male/female)	30 (58.82%) / 21 (41.18%)	35 (61.40%) / 22 (38.60%)	0.006	0.939	0.054	0.765
BMI (kg/m2)	28.18 ± 2.75	29.05 ± 3.21	1.514	0.133	-0.112	0.454
Diabetes	20 (39.22%)	28 (49.12%)	0.706	0.401	-0.273	0.041
Hypertension	42 (82.35%)	49 (85.96%)	0.062	0.803	-0.198	0.098
Cardiovascular disease	10 (19.61%)	20 (35.09%)	2.490	0.115	-0.287	0.035
Smoking history	15 (29.41%)	18 (31.58%)	0.001	0.972	-0.085	0.532
Drinking history	8 (15.69%)	11 (19.30%)	0.057	0.811	-0.044	0.793
Family history of CKD	7 (13.73%)	9 (15.79%)	0.001	0.976	-0.091	0.487

### Protein levels

The levels of LOX-1 (31.67 ± 4.23 vs. 38.01 ± 3.98), hs-cTnT (133.54 ± 12.79 vs. 158.21 ± 15.62), and NGAL (18.76 ± 3.21 vs. 24.08 ± 4.56) in patients with mild renal dysfunction were lower than those in patients with severe renal dysfunction, with statistically significant differences (*P* < 0.05) (Table 2). This suggests that these indicators may be related to the severity of renal dysfunction.

**Table 2:** Comparison of serum protein levels between the two groups

	Mild renal insufficiency group (*n* = 51)	Severe renal insufficiency group (*n* = 57)	t/X^2^	*P* value
LOX-1 (ng/mL)	31.67 ± 4.23	38.01 ± 3.98	7.992	< 0.001
hs-cTnT (pg/mL)	133.54 ± 12.79	158.21 ± 15.62	9.012	< 0.001
NGAL (ng/mL)	18.76 ± 3.21	24.08 ± 4.56	7.065	< 0.001

### Correlation analysis between serum levels of LOX-1, hs-cTnT, NGAL, and renal function

According to Table 3, correlation analysis reveals that serum LOX-1, hs-cTnT, and NGAL levels are positively correlated with renal function deterioration (*r* = 0.615, 0.655, 0.559, *P* < 0.001), indicating a significant association between LOX-1, hs-cTnT, NGAL levels and renal function deterioration. This suggests that serum LOX-1, hs-cTnT, and NGAL levels may serve as potential indicators for evaluating renal function impairment. Additionally, Pearson correlation analysis revealed significant positive correlations between the serum levels of the biomarkers: LOX-1 with hs-cTnT (*r* = 0.512, *P* < 0.001) and NGAL (*r* = 0.483, *P* < 0.001). Similarly, hs-cTnT showed a significant correlation with NGAL (*r* = 0.532, *P* < 0.001), suggesting interrelated pathological roles of these biomarkers in CKD progression.

**Table 3:** Correlation analysis between serum levels of LOX-1, hs-cTnT, MGA, and ACR

	With ACR (*r*)	R2	*P* value	With LOX-1 (*r*)	With hs-cTnT (*r*)	With NGAL (*r*)
LOX-1 (ng/mL)	0.615	0.378	< 0.001		0.512	0.483
hs-cTnT (pg/mL)	0.655	0.428	< 0.001	0.512		0.532
NGAL (ng/mL)	0.559	0.312	< 0.001	0.483	0.532	

### Diagnostic value of serum levels of LOX-1, hs-cTnT, NGAL for CKD

**Fig. 1:**
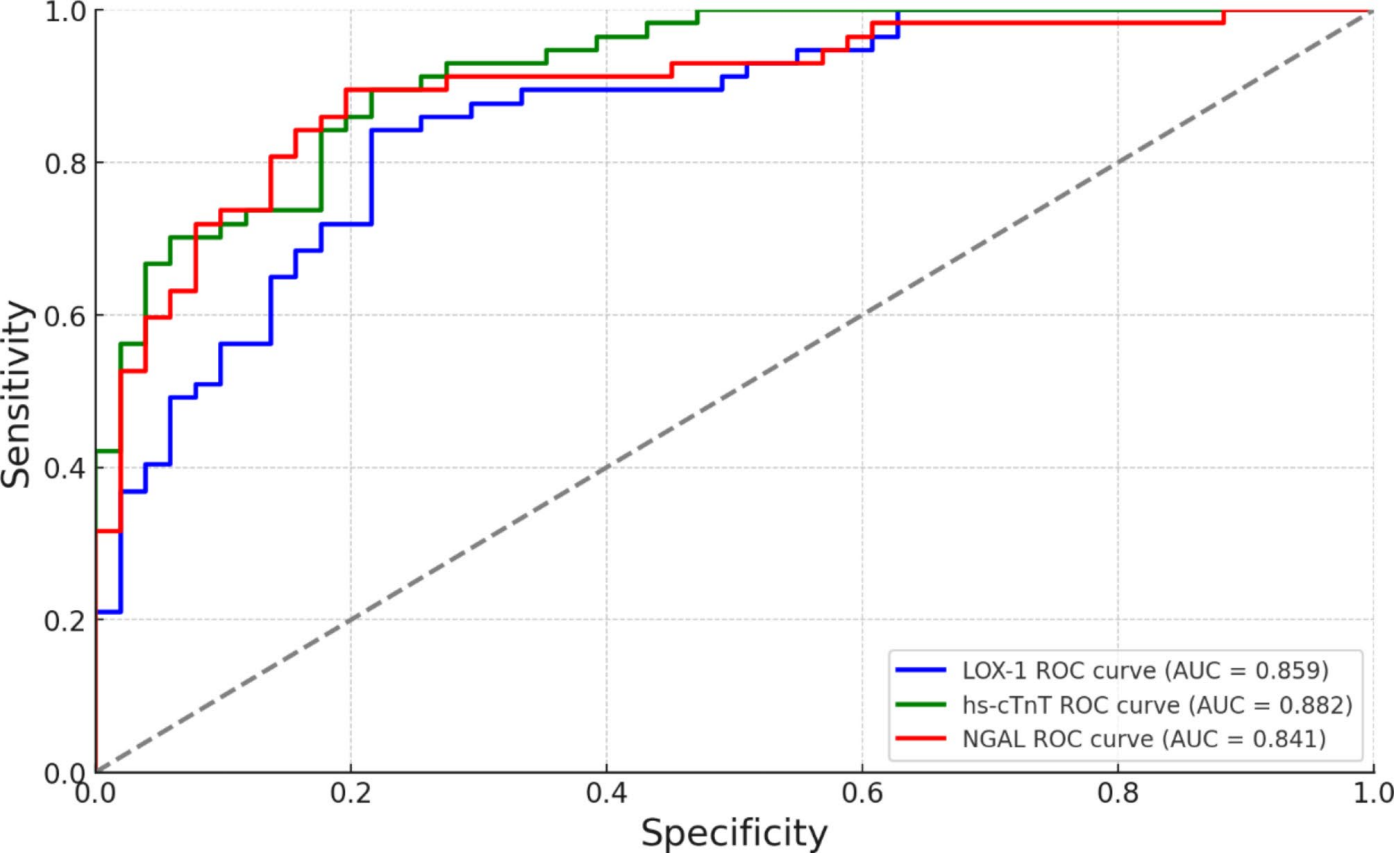
ROC Curves For LOX-1, Hs-CTnT, and NGAL. This ROC curve compares the predictive performance of serum levels of LOX-1, hs-cTnT, and NGAL in patients with mild and severe renal dysfunction. The levels of LOX-1 (31.67 ± 4.23 vs. 38.01 ± 3.98), hs-cTnT (133.54 ± 12.79 vs. 158.21 ± 15.62), and NGAL (18.76 ± 3.21 vs. 24.08 ± 4.56) were significantly lower in patients with mild renal dysfunction compared to those with severe renal dysfunction (*P* < 0.05), indicating a potential relationship between these biomarkers and the severity of renal dysfunction. The AUC values for each biomarker were 0.859 for LOX-1, 0.882 for hs-cTnT, and 0.841 for NGAL, suggesting that all three biomarkers have predictive value for assessing the progression of chronic kidney disease. Among them, hs-cTnT demonstrated the strongest predictive capability, followed by LOX-1 and NGAL

As shown in Table 4; Fig. 1, the AUC values for serum LOX-1, hs-cTnT, and NGAL levels were 0.859, 0.882, and 0.841, respectively. This indicates that serum LOX-1, hs-cTnT, and NGAL have certain predictive values for determining the progression of chronic kidney disease. Among them, hs-cTnT has the strongest predictive ability, followed by LOX-1 and NGAL.

**Table 4:** Predictive value of serum levels of LOX-1, hs-cTnT, NGAL for chronic kidney disease deterioration

Biomarker	Sensitivity	Specificity	AUC
LOX-1	0.632	0.941	0.859
hs-cTnT	0.754	0.902	0.882
NGAL	0.825	0.784	0.841

## Discussion

CKD is a serious disease characterized by structural and functional changes in the kidneys, which severely affects the quality of life of patients. The prevalence of CKD is about 8–16% in the world population [1, 2]. CKD is characterized by structural and functional changes in the kidneys, leading to a decline in renal function and a significant impact on patients’ quality of life [21]. Correlation analysis in this study revealed that age, diabetes, and cardiovascular disease were significantly negatively correlated with GFR, suggesting that these factors may accelerate the decline in kidney function (*P* < 0.05). Although the differences in these variables between the two groups were not statistically significant (*P* > 0.05), these factors may play a critical role in the onset and progression of CKD. Therefore, future research should further explore the long-term impact of these factors on kidney function, and strengthen the clinical management of related comorbidities to slow kidney function deterioration.

In recent years, there has been growing interest in identifying biomarkers that can aid in the diagnosis and management of CKD. Several studies have focused on the potential diagnostic value of serum levels of LOX-1, hs-cTnT, and NGAL in patients with CKD [22–24]. The LOX-1 receptor plays a crucial role in the pathogenesis of various diseases, including CKD. It has been implicated in the development and progression of renal injury through several mechanisms, including oxidative stress, inflammation, and endothelial dysfunction [25, 26]. The results of this study showed that the level of LOX-1 in patients with mild renal insufficiency (31.67 ± 4.23 vs. 38.01 ± 3.98) was lower than that in patients with severe renal insufficiency, and the level of LOX-1 was positively correlated with the severity of renal function (*r* = 0.615, *p* < 0.001). This suggests that the level of LOX-1 may be related to the severity of kidney injury, and its elevation can lead to glomerulosclerosis by acting on mesangial cells, ultimately causing kidney damage. Previous studies have demonstrated that LOX-1 is involved in the development of glomerulosclerosis, a common pathological feature of CKD. Activation of LOX-1 in mesangial cells can lead to increased oxidative stress, inflammation, and fibrosis, ultimately resulting in kidney damage [27, 28]. Therefore, the elevated level of LOX-1 in patients with severe renal insufficiency may indicate a more advanced stage of kidney injury.

In this study, the level of hs-cTnT in patients with severe renal insufficiency was significantly higher than that in patients with mild renal insufficiency, and the level of hs-cTnT was positively correlated with the deterioration of renal function (0.655, *P* < 0.001), suggesting a certain relationship between the level of hs-cTnT and the severity of kidney injury. Claudel, S.E. et al. also reached similar conclusions in their studies [29]. Overall, the results of this study highlight the importance of hs-cTnT as a potential diagnostic marker for assessing the severity of kidney injury in CKD patients. Further research is needed to validate these findings and explore the underlying mechanisms of hs-cTnT in kidney disease progression. This study further found a significant correlation between LOX-1, hs-cTnT, and NGAL, suggesting that these markers may not only be independent indicators of renal function deterioration but may also jointly participate in the pathophysiological process of CKD.

To further explore the diagnostic value of serum LOX-1, hs-cTnT, and NGAL levels, ROC analysis was conducted, and the results showed that the AUC of serum LOX-1, hs-cTnT, and NGAL levels were 0.859, 0.882, and 0.841, respectively. This indicates that the three markers have a certain predictive value for the worsening of chronic kidney disease. In summary, these findings highlight the potential of serum LOX-1, hs-cTnT, and NGAL as biomarkers for early diagnosis and monitoring of CKD progression. Importantly, this study suggests that elevated levels of these biomarkers not only indicate renal impairment but may also correlate with the underlying pathophysiological mechanisms of CKD, including oxidative stress and inflammation. Therefore, incorporating these biomarkers into routine clinical practice could enhance the assessment of renal function and improve patient management strategies in CKD. However, elevated LOX-1 and cTnT may be seen in patients with diabetes and CVD, and CKD patients are often combined with these diseases, which may lead to a decrease in the specificity of the markers. In the future, we need to further study the levels of markers in CKD patients combined with different disease states to improve the diagnostic value of LOX-1, hs-cTnT, and NGAL levels. Moreover, due to the small sample size in this study, the diagnostic results have certain limitations and further research with a larger sample size is needed. Secondly, the specific mechanisms by which LOX-1, hs-cTnT, and NGAL levels regulate CKD were not clarified in this study. This is also a future research direction to improve early diagnosis of CKD patients.

## Conclusion

Serum levels of LOX-1, hs-cTnT, and NGAL increased as renal insufficiency progressed from mild to severe in patients with CKD. These biomarkers were positively correlated with the decline in kidney function, reflecting kidney damage. ROC analysis demonstrated that these markers have significant predictive value for diagnosing CKD, with hs-cTnT showing the strongest diagnostic capability, followed by LOX-1 and NGAL. Although this study lacked a control group of healthy individuals, the elevated levels of these biomarkers in CKD patients suggest their potential use for early diagnosis and disease monitoring. However, further research is needed to validate these findings in larger cohorts and in combination with different comorbidities to ensure their specificity in CKD diagnosis.

## Data Availability

The data involved in the present study can be provided under reasonable request.
